# The Glymphatic System and Obesity: A Diffusion Tensor Imaging ALPS Study

**DOI:** 10.3390/biomedicines13112585

**Published:** 2025-10-22

**Authors:** Kang Min Park, Jin-Hong Wi, Bong Soo Park, Dong Ah Lee, Jinseung Kim

**Affiliations:** 1 Department of Neurology, Haeundae Paik Hospital, Inje University College of Medicine, Busan 48108, Republic of Korea; smilepkm@hanmail.net (K.M.P.); peony1003@gmail.com (D.A.L.); 2 Department of Thoracic and Cardiovascular Surgery, Busan Paik Hospital, Inje University College of Medicine, Busan 47392, Republic of Korea; wiccy@hanmail.net; 3 Department of Internal Medicine, Haeundae Paik Hospital, Inje University College of Medicine, Busan 48108, Republic of Korea; h00245@paik.ac.kr; 4 Department of Family Medicine, Busan Paik Hospital, Inje University College of Medicine, Busan 47392, Republic of Korea

**Keywords:** body mass index, glymphatic system, obesity

## Abstract

**Background**: Obesity is a known risk factor for neurodegenerative diseases, potentially due to impaired clearance of brain waste through the glymphatic system. While the association between obesity and brain dysfunction has been widely studied in populations with neurological conditions, it remains unclear whether glymphatic system function is already reduced in neurologically healthy individuals with obesity. This study aimed to investigate whether glymphatic system function, measured via the diffusion tensor image (DTI) analysis along the perivascular space (DTI-ALPS) index, differs according to obesity status in neurologically healthy adults. **Methods**: We retrospectively analyzed brain DTI data from 62 neurologically healthy participants stratified into underweight (<18.5 kg/m^2^), normal weight (BMI ≥ 18.5 and <23.0 kg/m^2^), overweight (BMI ≥ 23.0 and <25.0 kg/m^2^), and obese (≥25.0 kg/m^2^) groups based on the World Health Organization Asia-Pacific body mass index (BMI) criteria. Group differences were examined using Mann–Whitney U tests and analysis of covariance, after adjusting for age. **Results**: Participants with obesity had significantly lower DTI-ALPS index values (1.262 ± 0.150) compared to those in the normal weight (1.405 ± 0.168, *p* = 0.048) and overweight (1.423 ± 0.195, *p* = 0.029) categories, even after adjusting for age. The DTI-ALPS index was also significantly reduced in participants with obesity compared to participants in the BMI < 25 kg/m^2^ group (1.410 ± 0.176, *p* = 0.015). **Conclusions**: This study provides the first evidence that obesity is linked to reduced glymphatic system function, as reflected by lower DTI-ALPS index in neurologically healthy adults. These findings underscore the importance of maintaining a healthy body weight to preserve brain waste clearance mechanisms and may offer insights into early vulnerability to neurodegenerative changes.

## 1. Introduction

Obesity, commonly assessed using body mass index (BMI), has been widely recognized as a major risk factor for various neurodegenerative diseases [1]. Beyond its metabolic implications, obesity induces a chronic pro-inflammatory state that can disrupt peripheral and central physiological processes. High-fat diets, which often contribute to obesity, have been shown to impair lymphatic transport and alter lymph node architecture, indicating systemic inflammation and immune dysregulation [2]. In the central nervous system, obesity has been associated with increased perivascular inflammation, oxidative stress, and mitochondrial dysfunction [3]. These pathological processes may compromise the function of the glymphatic system—a brain-wide clearance pathway that facilitates the exchange of cerebrospinal fluid (CSF) and interstitial fluid (ISF). Disruption of this convective flow and CSF-to-ISF turnover may lead to glymphatic dysfunction.

Such impairment in glymphatic system function has been implicated in the pathogenesis of neurodegenerative diseases, including Parkinson’s disease (PD) and Alzheimer’s dementia, by reducing the clearance of metabolic waste and neurotoxic proteins from the brain parenchyma [4]. Therefore, exploring the link between obesity and glymphatic system function may provide novel insights into the mechanisms underlying neurodegeneration. Historically, it was believed that the brain, like other organs, managed protein waste exclusively through intracellular recycling mechanisms such as autophagy and proteasomal degradation [5]. However, this perspective was fundamentally altered with the introduction of the glymphatic system in 2012. The glymphatic system is now recognized as a brain-wide perivascular network that facilitates the exchange of CSF and ISF throughout the central nervous system, enabling the efficient clearance of soluble waste products and neurotoxic proteins from the brain [6].

To non-invasively evaluate the function of this system in vivo, recent studies have employed diffusion tensor imaging (DTI) magnetic resonance imaging (MRI), a technique sensitive to microstructural water movement in the brain [7,8]. The diffusion tensor image analysis along the perivascular space (DTI-ALPS) index is a diffusion MRI–derived metric designed to evaluate water diffusivity along perivascular pathways parallel to medullary veins. By comparing diffusivities parallel and perpendicular to these directions, the DTI-ALPS index provides an indirect, non-invasive measure of glymphatic transport efficiency. The DTI-ALPS method was developed based on the hypothesis that ISF in the white matter predominantly flows along the direction of medullary veins, which run parallel to the perivascular space [8]. This approach enables the indirect assessment of glymphatic system function by measuring water diffusivity aligned with the perivascular pathways. A major advantage of the DTI-ALPS technique is that it requires no administration of gadolinium-based contrast agents, utilizes a noninvasive method, and can be retrospectively applied to existing neuroimaging datasets. These features make the DTI-ALPS technique a practical and widely applicable tool for glymphatic system research.

Given prior evidence linking obesity to impaired brain homeostasis, we hypothesized that glymphatic system function, measured using the DTI-ALPS index, would differ according to whether individuals had obesity as defined by BMI. Accordingly, this study aimed to investigate the association between obesity and glymphatic system function in neurologically healthy individuals.

## 2. Methods

### 2.1. Study Participants and Clinical Assessment

This study was approved by the Institutional Review Board of Busan Paik Hospital (IRB No. 2025-07-023). Participants were neurologically healthy adults aged 19 years or older, none of whom had a history of any neurological or psychiatric disease that could influence brain morphology, and all demonstrated normal MRI findings on visual inspection [9]. For this analysis, only individuals who provided additional consent for the reuse of their existing neuroimaging data were enrolled. All participants were free from diagnosed neurological or chronic systemic illnesses and demonstrated preserved cognitive function, as evidenced by a perfect score (4/4) on the Korean version of the Abbreviated Mental Test—4 items [10]. The exclusion criteria included the presence of structural brain abnormalities on MRI, suboptimal diffusion image quality, or any medical conditions potentially affecting brain health.

### 2.2. Anthropometric Measurements and BMI Classification

Information on age and sex was retrieved from clinical records. Body weight and height were measured on the same day as MRI acquisition during participants’ hospital visits for health screening. Therefore, BMI represents the body composition status at the time of MRI data collection. Body weight and height were measured following standardized procedures, and BMI was computed by dividing body weight in kilograms by the square of height in meters (kg/m^2^). BMI classification was based on the World Health Organization’s (WHO) Asia-Pacific guidelines: underweight (<18.5 kg/m^2^), normal weight (BMI ≥ 18.5 and <23.0 kg/m^2^), overweight (BMI ≥ 23.0 and <25.0 kg/m^2^), and obesity (≥25.0 kg/m^2^) [11], and participants were grouped according to this classification.

### 2.3. MRI Data Acquisition

DTI MRI scans were obtained using a 3.0T MRI system (AchievaTx; Philips Healthcare, Best, the Netherlands) with a 32-channel head coil. The diffusion protocol included 32 non-collinear directions with b-values of 0 and 1000 s/mm^2^, a repetition time of 8620 ms, an echo time of 85 ms, and a flip angle of 90°. Other scan parameters included: 2.25 mm slice thickness, 120 × 120 acquisition matrix, 240 × 240 mm field of view, and a parallel imaging factor (SENSE) of 2. The phase-encoding direction was anterior-to-posterior, and fat suppression was applied in the posterior direction.

### 2.4. DTI-ALPS Index Calculation

The procedure for calculating the DTI-ALPS index is illustrated in Supplementary Figure S1. Preprocessing and reconstruction of diffusion data were performed using diffusion spectrum imaging (DSI) Studio software (Version 2021 May, http://dsi-studio.labsolver.org, accessed on 14 August 2025). Diffusion data were reconstructed using the diffusion tensor model in DSI Studio. The fractional anisotropy (FA) threshold was 0.2, angular threshold 45°, step size 1 mm, diffusion sampling length ratio 1.25, and no spatial smoothing was applied. Preprocessing included eddy-current and motion correction, skull stripping, and mask generation using standard DSI Studio defaults. A 3 × 3 voxel (approximately 6 × 6 × 2 mm^3^) region of interest (ROI) was selected to balance anatomical specificity and signal stability, consistent with previous ALPS studies. Averaging within this small region reduces voxel-level noise while maintaining sensitivity to local microstructural features. ROIs were restricted to the left hemisphere to ensure methodological consistency and minimize hemispheric variability due to potential lateral dominance or partial-volume effects. Prior methodological validation has shown minimal asymmetry in healthy individuals. Projection-fiber ROIs were located in the corona radiata adjacent to the lateral ventricle, while association-fiber ROIs were placed in the superior longitudinal fasciculus on the same axial plane. Placement followed the anatomical guidance described in prior methodological literature and was visually confirmed on color-coded FA maps. The pipeline included correction of eddy current and susceptibility-induced distortions, skull stripping, mask generation, and diffusion tensor modeling.

Using a standardized protocol, 3 × 3 voxel-sized regions of interest were placed in areas representing projection and association fibers in the left hemisphere. Diffusivity values along the *x*-, *y*-, and *z*-axes were extracted within these regions. For each fiber type, the voxel with the highest directional orientation along the *x*-axis was selected. The DTI-ALPS index was computed using the following formula [12]:

$$DTI\text{-}ALPS\;index\;=\;\frac{Mean(D_{xx}^{proj},\;D_{xx}^{assoc})}{Mean(D_{yy}^{proj},\;D_{zz}^{assoc})}$$

where $D_{xx}^{proj}$ and $D_{xx}^{assoc}$ denote *x*-axis diffusivity in projection and association fibers, respectively, and $D_{yy}^{proj}$ and $D_{zz}^{assoc}$ represent *y*- and *z*-axes diffusivity in the same fibers. The procedure for DTI-ALPS index calculation is illustrated in Figure S1.

### 2.5. Statistical Analyses

All statistical analyses were conducted using Python 3.10 (Python Software Foundation, Wilmington, DE, USA) with the following libraries: pandas (v1.5.3) for data handling, scipy (v1.10.1) for non-parametric testing, statsmodels (v0.13.5) for regression modeling and analysis of covariance (ANCOVA), and matplotlib and seaborn for data visualization. Descriptive statistics were used to summarize participants’ characteristics. Continuous variables are presented as mean ± standard deviation (SD), and categorical variables as numbers and percentages. Group differences in basic participant characteristics (Table 1) were assessed using one-way analysis of variance for continuous variables (age, weight, height, BMI) and the chi-square test for the categorical variable (sex). For DTI-derived diffusivity metrics and the DTI-ALPS index (Table 2), participants were divided into two groups: participants without (BMI < 25 kg/m^2^) and with obesity (BMI ≥ 25 kg/m^2^). Differences between these two groups were assessed using both independent *t*-tests and Mann–Whitney U tests. Results are reported as mean (standard deviation) for each group along with associated *p*-values from both statistical tests. Additionally, Pearson’s correlation coefficient and Spearman’s rank correlation coefficient were calculated to assess the relationship between the DTI-ALPS index and continuous variables, including BMI and age. Two-sided tests were used, and *p* < 0.05 was considered statistically significant.

To evaluate DTI-ALPS index differences more specifically, the following statistical approaches were applied:

Unadjusted group comparisons (Mann–Whitney U test)

Given the small sample sizes and potential deviations from normality, non-parametric Mann–Whitney U tests were performed to compare the DTI-ALPS index between participants with obesity (BMI ≥ 25 kg/m^2^) and the following BMI subgroups:

Participants who were of normal weight (BMI ≥ 18.5 and <23.0 kg/m^2^)

Participants who were overweight (BMI ≥ 23.0 and <25.0 kg/m^2^)

Participants without obesity (BMI < 25 kg/m^2^)

Because the underweight group contained only two participants, non-parametric Mann–Whitney U tests were used for pairwise group comparisons (e.g., obesity vs. normal weight, obesity vs. overweight). The Kruskal–Wallis test was not applied because highly unbalanced subgroup sizes would compromise the validity of non-parametric multi-group testing. For two-group comparisons, participants were classified as with obesity (BMI ≥ 25 kg/m^2^) or without obesity (BMI < 25 kg/m^2^), the latter combining normal-weight and overweight individuals. This dichotomization aligns with the WHO Asia–Pacific obesity threshold, where metabolic risk substantially increases, and also ensures adequate statistical power for between-group analyses.

#### Age-Adjusted Group Comparisons (ANCOVA)

To adjust for the potential confounding effect of age, ANCOVA was conducted using an ordinary least squares regression model. The DTI-ALPS index was entered as the dependent variable, BMI group as the categorical independent variable, and age as the covariate. Separate ANCOVA models were constructed for the following group comparisons:

- Participants with obesity vs. participants who were of normal weight;
- Participants with obesity vs. participants who were overweight;
- Participants with obesity vs. participants without obesity.

Adjusted group means were estimated, and statistical significance was evaluated based on the group effect (*p* < 0.05).

## 3. Results

### 3.1. Participants’ Characteristics by BMI Category

The demographic and anthropometric characteristics of participants stratified by BMI category are summarized in Table 1. A total of 62 participants were included and categorized into four groups based on the WHO Asia-Pacific criteria: participants with underweight (*n* = 2), normal weight (*n* = 29), overweight (*n* = 21), and obesity (*n* = 10). There were no significant differences in age among the groups (*p* = 0.819). The proportion of male participants differed significantly across groups (*p* < 0.001), ranging from 0% (*n* = 0) in the underweight group to 57.1% (*n* = 12) in the overweight group. Significant differences were observed in body weight (*p* = 0.021), height (*p* < 0.001), and BMI (*p* = 0.042), with progressive increases from underweight to people with obesity.

### 3.2. DTI-Derived Diffusivity Measures and ALPS Index

Table 2 summarizes the DTI-derived diffusivity values and DTI-ALPS index between participants with and without obesity. The DTI-ALPS index was significantly lower in participants with obesity (1.262 ± 0.150) compared to those without obesity (1.410 ± 0.176; *p* = 0.0361, Mann–Whitney U test). Among the diffusivity measures, projection fiber Dxx was significantly lower in participants with obesity (5.430 ± 0.340) than in those without obesity (6.028 ± 0.538; *p* = 0.0009). Although association fiber Dyy appeared lower in participants with obesity (10.241 ± 0.912) than in those without obesity (10.740 ± 0.870), the difference was not statistically significant (*p* = 0.1281). No statistically significant differences were observed in the remaining parameters, including projection Dyy and Dzz, association Dxx and Dzz, and all subcortical fiber diffusivities (all *p* > 0.05, Mann–Whitney U test).

### 3.3. Unadjusted Comparisons (Mann−Whitney U Tests)

**Figure 1:**
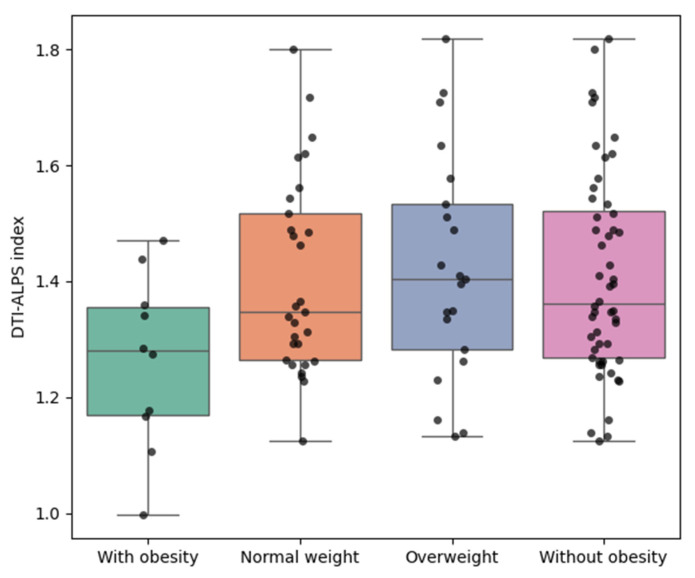
Unadjusted group comparisons of DTI-ALPS index by BMI category.

Although BMI was used for group stratification, comparing DTI-ALPS indices across BMI-defined categories allows evaluation of non-linear changes in glymphatic function rather than reiterating BMI differences per se. To explore unadjusted group differences in glymphatic function, we conducted non-parametric Mann–Whitney U tests due to the relatively small sample sizes and potential violations of normality assumptions. As illustrated in Figure 1, participants with obesity (BMI ≥ 25.0 kg/m^2^) exhibited significantly lower DTI-ALPS index values compared to all three comparison groups. Compared to the individuals with normal weight (BMI ≥ 18.5 and <23.0 kg/m^2^), participants with obesity showed a significantly reduced DTI-ALPS index (mean ± SD: 1.262 ± 0.150 vs. 1.405 ± 0.168; U = 83.000, *p* = 0.0479). Similarly, when compared to individuals in the overweight group (BMI 23.0–24.9 kg/m^2^), the difference approached statistical significance (1.262 ± 0.150 vs. 1.423 ± 0.195; U = 59.000, *p* = 0.0545). Finally, when compared to individuals without obesity (BMI < 25 kg/m^2^), the difference remained statistically significant (1.262 ± 0.150 vs. 1.410 ± 0.176; U = 150.000, *p* = 0.0361).

Boxplots illustrating the distribution of the DTI-ALPS index across four BMI-defined groups:

- Participants with obesity (BMI ≥ 25.0 kg/m^2^);
- Participants who were normal weight (BMI ≥ 18.5 and < 23.0 kg/m^2^);
- Participants who were overweight (BMI ≥ 23.0 and < 25.0 kg/m^2^);
- Participants without obesity (BMI < 25.0 kg/m^2^).

Each dot represents an individual participant. Prior to age adjustment, participants with obesity exhibited lower DTI-ALPS index values compared to the other groups. DTI-ALPS: diffusion tensor image analysis along the perivascular space; BMI: body mass index.

### 3.4. Age-Adjusted Comparisons (ANCOVA)

**Figure 2:**
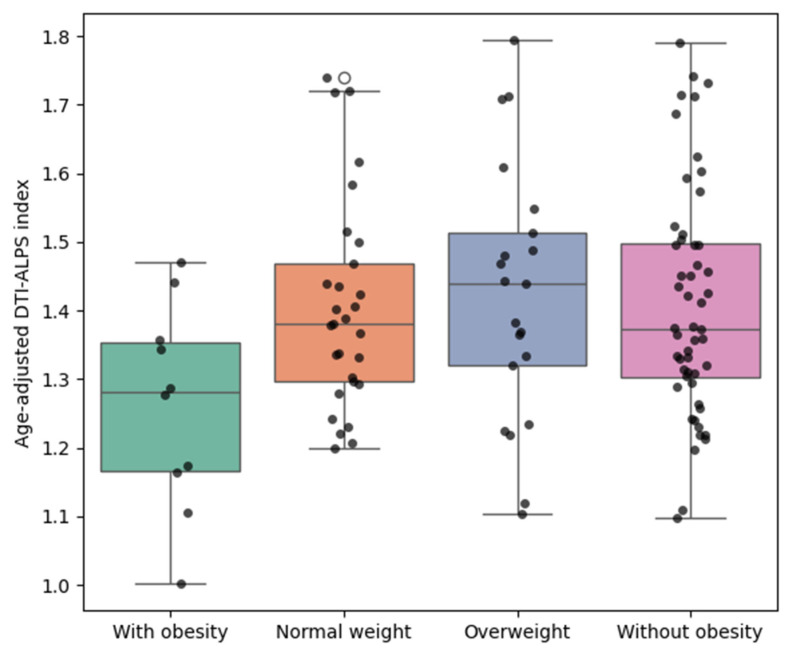
Age-adjusted group comparisons of DTI-ALPS index by BMI category. Boxplots illustrating the distribution of age-adjusted DTI-ALPS index values across the same four BMI-defined groups of participants shown in Figure 1.

To account for age-related confounding effects on glymphatic function, we performed ANCOVA, using BMI group as the independent variable and age as a covariate. As shown in Figure 2, the DTI-ALPS index remained significantly lower in participants with obesity across all comparisons after adjusting for age. In the comparison between obesity and people with normal weight, a significant group effect was observed (F(1,36) = 5.68, *p* = 0.0226), with age showing a marginal association with the DTI-ALPS index (F(1,36) = 4.11, *p* = 0.0501). Similarly, when compared with the overweight group, the DTI-ALPS index remained significantly reduced in participants with obesity (F(1,28) = 5.33, *p* = 0.0285), while age was not a significant covariate (F(1,28) = 1.17, *p* = 0.2882). When comparing participants with obesity to those without obesity (BMI < 25 kg/m^2^), the group difference remained statistically significant (F(1,59) = 6.24, *p* = 0.0153), and age was also significantly associated with the outcome (F(1,59) = 4.96, *p* = 0.0298). In all models, adjusted mean DTI-ALPS index values for participants with obesity remained consistently lower than those of the comparison groups.

Values represent estimated marginal means derived from analysis of covariance (ANCOVA) controlling for age. Each dot represents an individual participant. DTI-ALPS: diffusion tensor image analysis along the perivascular space; BMI: body mass index.

### 3.5. Correlation Between DTI-ALPS Index, BMI, and Age

Correlations were calculated between the DTI-ALPS index, BMI, and age. The correlation between BMI and DTI-ALPS index was Pearson’s r = –0.123 (*p* = 0.3428) and Spearman’s ρ = –0.116 (*p* = 0.3676). The correlation between age and DTI-ALPS index was Pearson’s r = –0.274 (*p* = 0.0312) and Spearman’s ρ = –0.274 (*p* = 0.0314).

## 4. Discussion

In this study, we investigated the association between obesity and glymphatic system function using the DTI-ALPS index in neurologically healthy adults. Our findings demonstrated that participants with obesity (BMI ≥ 25 kg/m^2^) exhibited significantly lower DTI-ALPS index values compared to both participants with normal weight (BMI ≥ 18.5 and <23.0 kg/m^2^) and those without obesity (BMI < 25 kg/m^2^). These differences remained statistically significant even after adjusting for age, suggesting that reduced glymphatic function is independently associated with obesity rather than being solely attributable to age-related decline.

The glymphatic system, a recently characterized perivascular network, plays a crucial role in maintaining brain homeostasis by clearing interstitial solutes, including proteins and toxic metabolites [13]. The DTI-ALPS index, derived from DTI, quantifies water diffusivity along perivascular spaces and serves as an indirect marker of glymphatic function. A reduced DTI-ALPS index, as observed in individuals with obesity, may reflect impaired glymphatic clearance and the accumulation of neurotoxic substances, potentially increasing susceptibility to neuroinflammation and neurodegeneration. A previous study reported that the DTI-ALPS index declines with age and mediates the relationship between aging and cognitive performance, with a stronger effect observed in older adults [14]. Similarly, our results demonstrated a significant negative correlation between age and the DTI-ALPS index. This finding reinforces the evidence that glymphatic function diminishes progressively with aging, even in neurologically healthy individuals. Our results are consistent with a previous study on patients with PD, which reported a stepwise decline in the DTI-ALPS index with increasing BMI (normal weight > overweight > obesity), with individuals with obesity showing significantly lower DTI-ALPS index values [15]. While that study was limited to participants with neurodegenerative disease, our findings extend this association to neurologically healthy adults. This distinction is important, as it suggests that glymphatic dysfunction related to obesity may begin in the absence of any clinical neurological disorders, and that even early or subclinical obesity-related changes in glymphatic clearance can be detected in otherwise healthy individuals.

Obesity has been increasingly implicated as a risk factor for cognitive impairment and structural brain changes. Mid-life obesity is a well-established risk factor for Alzheimer’s disease and vascular dementia [16,17]. Even in people without neurological disease, elevated BMI is associated with poorer episodic and working memory performance [18,19,20], as well as reduced functional activity in memory-related brain regions such as the hippocampus, angular gyrus, and prefrontal cortex [21,22]. Neuroimaging studies further demonstrate that higher BMI correlates with reduced total brain volume [23,24], gray matter atrophy in the frontal, temporal, and occipital cortices, and the hippocampus and thalamus [25], along with decreased white matter integrity [26] and regional hypoperfusion in the prefrontal cortex [27]. One plausible biological mechanism involves chronic low-grade inflammation mediated by adipokines. In people with obesity, adipose tissues secrete elevated levels of pro-inflammatory cytokines, including tumor necrosis factor-alpha, interleukin (IL)-6, IL-1β, C-reactive protein, and plasminogen activator inhibitor-1 [28,29], along with reduced levels of adiponectin, which normally exerts anti-inflammatory and neuroprotective effects [30,31]. This pro-inflammatory state may disrupt the blood–brain barrier, damage white matter, and impair glymphatic clearance [32,33]. Inflammatory signals through the choroid plexus and direct effects on brain structures such as the hippocampus, cerebral cortex, brainstem, and amygdala have also been implicated [34,35].

Additionally, metabolic dysregulation observed in people with obesity can impair synaptic plasticity and lead to neuronal death, potentially contributing to the development of neurodegenerative diseases such as PD, Alzheimer’s disease, and multiple sclerosis [36,37]. These pathological changes can precede clinical symptoms and may begin during mid-life, supporting the importance of early detection and monitoring of glymphatic function in at-risk individuals.

In the context of Asian populations, including Koreans, the risks of metabolic and cardiovascular complications appear to be at lower BMI thresholds than in Western populations [11,38]. To reflect this, the WHO Asia–Pacific classification defines overweight as BMI ≥ 23 kg/m^2^ and obesity as BMI ≥ 25 kg/m^2^ [16]. Our study, using this regionally appropriate definition, demonstrated that even at these lower BMI cut-offs, people with obesity exhibited significantly reduced DTI-ALPS index values. This supports the relevance of ethnicity-specific obesity criteria when evaluating the impact of adiposity on brain health and glymphatic function.

Taken together, our results provide neuroimaging-based evidence that obesity may be detrimental to glymphatic activity, even in neurologically healthy adults. The observed reduction in DTI-ALPS index values in people with obesity, both before and after adjusting for age, suggests a robust association between excess body weight and impaired perivascular waste clearance.

To our knowledge, this is the first study to directly examine the relationship between obesity—defined by BMI—and glymphatic function as measured by the DTI-ALPS index. By focusing on neurologically healthy individuals, our study isolates the potential contribution of obesity to glymphatic dysfunction, independent of overt neurological disease. This offers new insights into how adiposity may affect brain clearance mechanisms and supports the use of the DTI-ALPS index as a biomarker for early brain vulnerability related to obesity.

Despite these strengths, this study had several limitations. The relatively small sample size and single-center retrospective design may limit the generalizability of findings and preclude causal inference. The use of self-reported data for medical history also introduces the possibility of misclassification or underestimation of comorbid conditions. In addition, the relatively small number of participants in the overweight category may have reduced the statistical power to detect differences in this subgroup. The total sample size was limited (*n* = 62), and particularly the underweight subgroup (*n* = 2) restricted multi-group comparisons. Consequently, the findings should be interpreted cautiously and confirmed in larger, prospective cohorts. The DTI-ALPS index reflects diffusion anisotropy along perivascular structures; however, the recent literature has suggested that it may represent microstructural alterations around perivascular spaces rather than direct measurements of CSF flow dynamics [39]. Therefore, the observed changes should be interpreted as indicative of perivascular diffusivity differences associated with obesity rather than definitive proof of altered glymphatic flow.

Nonetheless, by directly quantifying the association between BMI-defined obesity and a neuroimaging marker of glymphatic function, our study provides a foundation for future longitudinal investigations exploring how excess adiposity may contribute to brain aging, impaired waste clearance, and neurodegeneration. These findings underscore the importance of maintaining a healthy body weight to preserve brain health and suggest that even modest elevations in BMI—particularly in Asian populations—may have significant implications for glymphatic function and long-term neurological outcomes.

A recent study reported no significant difference in the DTI-ALPS parameter among healthy controls, obese individuals without food addiction, and obese individuals with food addiction, but suggested a declining trend from healthy controls to obese participants with food addiction [40]. In contrast, our study demonstrated a statistically significant reduction in the DTI-ALPS index among adults with obesity. This difference may reflect age-related or severity-dependent variations in glymphatic function, indicating that measurable impairment becomes evident in older or more metabolically affected individuals.

## 5. Conclusions

This study provides novel evidence that obesity, defined by BMI ≥25 kg/m^2^, is associated with reduced glymphatic system function as measured by the DTI-ALPS index, even in neurologically healthy participants. The observed decline in glymphatic system function in participants with obesity—independent of age—suggests that excess adiposity may impair brain waste clearance mechanisms. These findings suggest that the DTI-ALPS index can serve as a valuable tool for identifying early glymphatic system dysfunction in people with obesity and highlight the significance of obesity prevention in preserving neurological health.

## Figures and Tables

**Table 1:** Basic characteristics of participants by BMI category.

	Participants with Underweight	Participants with Normal Weight	Participants with Overweight	Participants with Obesity	*p*-Value
Age (years)	34.5 (10.6)	42.9 (12.7)	44.3 (17.2)	44.3 (12.6)	0.819
Male, *n* (%)	0 (0.0%)	7 (24.1%)	12 (57.1%)	2 (20.0%)	<0.001
Weight (kg)	46.1 (3.7)	54.4 (7.0)	67.1 (8.3)	81.7 (17.2)	0.021
Height (cm)	164.9 (0.1)	160.3 (8.0)	167.0 (9.9)	170.2 (13.7)	<0.001
BMI (Kg/m^2^)	16.96 (1.39)	21.11 (1.08)	23.95 (0.58)	27.87 (2.19)	0.042

Values are presented as mean (standard deviation) or number (%); *p*-values were calculated using one-way analysis of variance (ANOVA) for continuous variables and the chi-square test for categorical variables. BMI categories: Participants with underweight (BMI < 18.5 kg/m^2^), Participants with normal weight (BMI ≥ 18.5 and <23.0 kg/m^2^), Participants with overweight (BMI ≥ 23.0 and <25.0 kg/m^2^), Participants with obesity (≥25.0 kg/m^2^). BMI: body mass index.

**Table 2:** Differences in diffusivity measures and DTI-ALPS index between people with obesity and people without obesity.

	Participants Without Obesity, N = 52 (83.9%)	Participants with Obesity, N = 10 (16.1%)	*p*-Value (*t*-Test)	*p*-Value (Mann–Whitney U Test)
Projection fiber				
Dxx (×10^−4^)	6.028 (0.538)	5.430 (0.340)	0.0002	0.0009
Dyy (×10^−4^)	5.118 (0.901)	5.185 (0.780)	0.8136	0.612
Dzz (×10^−4^)	9.708 (0.931)	9.593 (0.601)	0.6201	0.4729
Association fiber				
Dxx (×10^−4^)	6.556 (0.718)	6.241 (0.691)	0.2118	0.2795
Dyy (×10^−4^)	10.740 (0.870)	10.241 (0.912)	0.1358	0.1281
Dzz (×10^−4^)	3.911 (0.507)	4.181 (0.791)	0.3220	0.1653
Subcortical fiber				
Dxx (×10^−4^)	10.768 (1.166)	10.711 (1.202)	0.8916	0.9924
Dyy (×10^−4^)	7.113 (1.329)	6.301 (1.030)	0.0461	0.062
Dzz (×10^−4^)	5.924 (1.543)	5.543 (0.754)	0.2445	0.6529
DTI-ALPS index	1.410 (0.176)	1.262 (0.150)	0.0147	0.0361

Values are presented as mean (standard deviation). Group comparisons between people with obesity and people without obesity were performed using both independent *t*-tests and Mann–Whitney U tests. BMI: body mass index; DTI-ALPS: diffusion tensor image analysis along the perivascular space.

## Data Availability

The original contributions presented in this study are included in the article/Supplementary Material. Further inquiries can be directed to the corresponding author(s).

## Supplementary Materials

The following supporting information can be downloaded at: https://www.mdpi.com/article/10.3390/biomedicines13112585/s1. Figure S1: Schematic illustration of the DTI-ALPS index calculation method.
