# Supplementary material for: The Glymphatic System and Obesity: A Diffusion Tensor Imaging ALPS Study
Source: Biomedicines. 2025 Oct 22;13(11):2585. doi: 10.3390/biomedicines13112585 (PMC12650082; doi:10.3390/biomedicines13112585)
Supplement: Supplementary file 1 [file biomedicines-13-02585-s001.zip › biomedicines-3885405-supplementary.pdf]

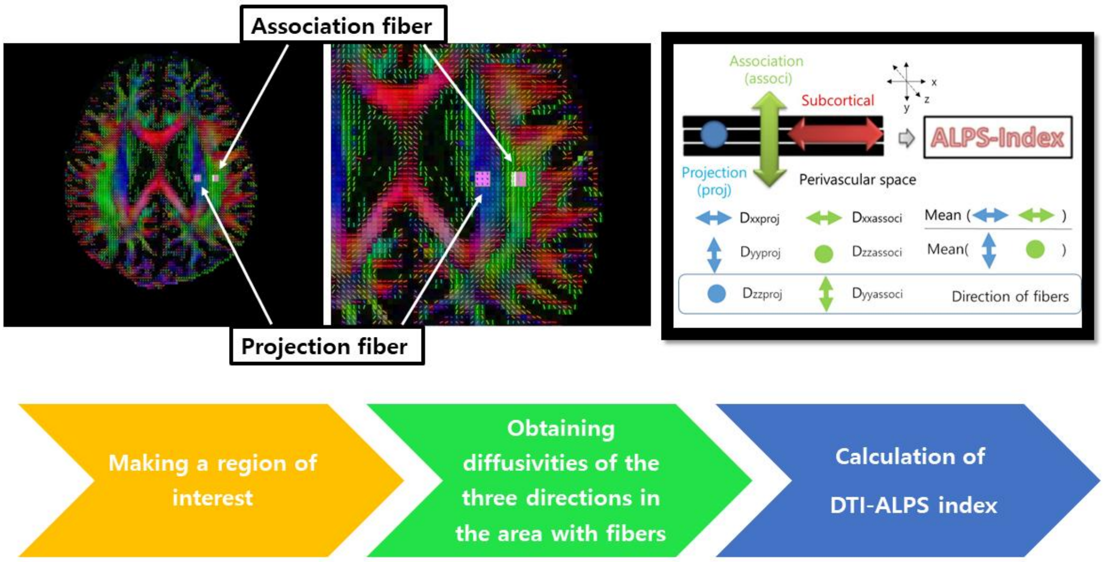

Figure S1. Schematic illustration of the DTI-ALPS index calculation method. Left: Regions of interest (ROIs) were placed on projection and association fibers in the left hemisphere. Middle: Diffusivities along the x, y, and z directions (Dxx, Dyy, Dzz) were extracted for each fiber type. Right: The DTI-ALPS index was calculated as the ratio of diffusivity along the perivascular space (x-direction) to those orthogonal to the fibers. The stepwise process is visualized below, from ROI selection to DTI-ALPS index computation.
